# V1/V2-directed antibodies elicited in RV144 vaccinees bind to a structurally polymorphic site

**DOI:** 10.1186/1742-4690-9-S2-P107

**Published:** 2012-09-13

**Authors:** JS McLellan, J Gorman, M Bonsignori, K Hwang, H Liao, S Rerks-Ngarm, S Nitayaphan, NL Michael, JH Kim, BF Haynes, PD Kwong

**Affiliations:** 1 National Institutes of Health, Bethesda, MD, USA; 2 The Duke Human Vaccine Institute, Durham, NC, USA; 3 Department of Disease Control, Bangkok, Thailand; 4 Department of Retrovirology and AFRIM, Bangkok, Thailand; 5 U.S. Military HIV Research Program, Rockville, MD, USA

## Background

An immune correlates study of the RV144 vaccine trial demonstrated that elicitation of V1/V2-directed antibodies was inversely associated with infection risk. In addition, a sieve analysis of breakthrough infections identified residue 169 in the V2 loop as a site of immune pressure. Antibodies CH58 and CH59 were isolated from RV144 vaccinees and were shown to neutralize some Tier 1 isolates.

## Methods

The epitopes for CH58 and CH59 were identified by peptide-mapping and alanine-scanning. Antigen-binding fragments (Fabs) were generated by proteolysis, and crystal structures of the CH58 and CH59 Fabs in complex with a linear V2 peptide were determined to 1.7 and 1.5 Å, respectively. Surface plasmon resonance was used to determine the kinetics of antibody binding to recombinant env-derived proteins.

## Results

The crystal structures reveal that CH58 recognizes V2 residues 167-176 as an α-helix and residues 177-181 as an extended coil. In contrast, CH59 recognizes residues 168-173 as coil, with residues 174-176 as a short 3_10_ helix. Both antibodies form hydrogen bond or salt-bridge interactions with the side chain of Lys169, the imputed site of immune pressure. The conformations of V2 recognized by CH58 and CH59 differ markedly from the β-strand conformation recognized by the broadly neutralizing antibody PG9. All three antibodies bound with high affinity to the same gp120 protein, suggesting that V2 residues 167-176 can adopt multiple conformations on a shed gp120.

## Conclusion

Since PG9 is broadly neutralizing and recognizes V2 as a β-strand, and because CH58 and CH59 neutralize only some Tier 1 isolates and recognize alternative conformations of V2, these data suggest that the β-strand conformation of V2 may be favored in the viral spike, whereas alternative V2 conformations may be favored on shed gp120s. If true, then vaccine immunogens may need to have the V1/V2 region stabilized in the β-strand conformation in order to elicit broadly neutralizing antibodies.

